# Endoscopic supracerebellar transtentorial approach for amygdalohippocampectomy (ESTAH): an anatomic study of the minimally invasive keyhole technique

**DOI:** 10.1007/s10143-025-03494-1

**Published:** 2025-04-16

**Authors:** Serdar Rahmanov, Romel Corecha Santos, Mohammadmahdi Sabahi, Diego Morales Roccuzzo, Bhavika Gupta, Shadi Bsat, Abdulrahman Albakr, Brandon Kaye, Raphael Bastianon Santiago, Hamid Borghei-Razavi, Badih A. Adada

**Affiliations:** 1 Department of Neurological Surgery, Pauline Braathen Neurological Center, Cleveland Clinic Florida, Weston, FL USA; 2 Division of Pediatric Neurosurgery, Department of Neurosurgery, University of Alabama at Birmingham, Birmingham, AL USA; 3 Department of Neurosurgery, Allegheny General Hospital, Pittsburgh, PA USA; 4 Department of Surgery, Division of Neurosurgery, King Saud University, Riyadh, Saudi Arabia; 5 Dr. Kiran C. Patel College of Allopathic Medicine, Nova Southeastern University, Fort Lauderdale, FL USA

**Keywords:** Endoscopic approach, Keyhole approach, Supracerebellar transtentorial approach, Amygdalahipocamectomy, Microscopic fiber dissection, Microsurgical anatomic study

## Abstract

**Objective:**

The mediobasal temporal region (MTR) is a complex neurosurgical target due to its deep location and proximity to critical neurovascular structures. Conditions such as mesial temporal sclerosis frequently involve the MTR, often leading to epilepsy. Traditional approaches, including anterior lobectomy and transcortical amygdalohippocampectomy, are effective but carry risks such as visual field deficits from optic radiation damage. This study evaluates the feasibility and effectiveness of the endoscopic supracerebellar transtentorial approach (ESTAH) as a minimally invasive alternative, emphasizing neurovascular preservation and minimizing complications.

**Methods:**

Four formalin-fixed, silicon-injected cadaver heads and ten human cadaver brains prepared using the Klingler method were dissected to study MTR anatomy. ESTAH was performed using 0° and 30° endoscopes and neuronavigation. Key anatomical landmarks, including the tentorial angle and the posterior fossa dura-to-hippocampal uncus distance, were measured to assess surgical precision and safety.

**Results:**

The endoscope provided precise visualization and resection of the hippocampus, amygdala, and parahippocampal gyrus with minimal disruption to optic radiations and adjacent white matter tracts. Critical neurovascular structures, such as the internal carotid artery, middle cerebral artery branches, posterior cerebral artery, and cranial nerves III and IV, were preserved. The average dura-to-uncus distance was 78.15 mm, and the average tentorial angle was 104.1°.

**Conclusion:**

The ESTAH is a feasible, minimally invasive alternative for amygdalohippocampectomy, offering excellent visualization and reduced risk of complications. This approach has the potential to improve surgical outcomes and minimize morbidity in MTR surgeries.

## Introduction

The meidobasal temporal region (MTR) presents significant challenges for neurosurgeons worldwide and demands precise and deliberate surgical planning; rather, it should be deeply scrutinized and systematically studied. This is due to its deep-seated location in the brain and the critical neurovascular structures that surround it. Consequently, various techniques have been developed over the past eighty years for operating in this area. The primary indication for an amygdalohippocampectomy is epilepsy control. Although Spencer et al. proposed an anterior lobectomy in conjunction with the amygdalohippocampectomy as a viable surgical method [1, 2], most neurosurgeons opt for a selective amygdalohippocampectomy via a temporal transcortical approach, initially described by Niemeyer [3]. Other prevalent techniques include the transsylvian corridor, as advocated by Yasargil [4], or a subtemporal approach, popularized by Hori et al. [5]. Each technique has its own advantages and limitations.

Recent studies indicate promising outcomes regarding epilepsy control, with up to 70% of patients experiencing a reduction in seizure rates [6]. The mortality rate varies from 0 to 3.5%, while cumulative morbidity rates can reach up to 88% if complications like visual field disturbances are included [7]. However, visual field deficits are often considered separate complications, with deficits ranging from 54–87% [8].

To lessen visual field issues following amygdalohippocampectomy, a technique was introduced by Türe et al. in 2012 [9]. This method is characterized as a microscopic paramedian supracerebellar–transtentorial (PST) approach, initially described by Yasargil [10]. It aims to establish a direct corridor to the MTR without cortical violation. However, there were concerns about resecting or damaging the amygdala and piriform cortex through this corridor. Nonetheless, this approach is an excellent option for accessing more posterior lesions. Despite the original technique involving endoscopic-assisted surgery, the endoscope plays a secondary role and is reserved for later stages of the procedure.

The purpose of this study is to investigate the application of the endoscopic supracerebellar transtentorial approach (ESTAH) for amygdalohippocampectomy. Specifically, we aim to demonstrate the practical effectiveness of this minimally invasive technique in accessing the MTR [11]. Additionally, this anatomical study will provide a detailed overview of the MTR’s anatomy and assess the feasibility of performing a complete ESTAH, addressing the technical nuances associated with the procedure.

## Materials and methods

### Cadaveric preparation

The study was performed in the Cleveland Clinic Florida Skull Base Microsurgery Laboratory on four formalin-fixed, alcohol-preserved, silicon-injected cadaver heads and 10 human cadaver brains that had been fixed using the Klingler method for step-by-step microsurgical white matter fiber dissection [12]. 

Dissections were carried out using a rod lens endoscope (4 mm, 18 cm, Hopkins II, 0°, and 30° (Karl Storz, Tuttlingen, Germany) and a Leica M320 microscope (Leica Microsystems, Wetzlar, Germany) with a magnification range of 6× to 40×. All dissections were performed bilaterally with standard microsurgical and endoscopic instrumentation. Two-dimensional and Three-dimensional images were taken at each dissection stage using a Nikon^®^ D750 camera (SS-MS1), paired with a Nikon AF-S Macro NIKKOR Lens 105 mm 1:2.8G ED and zero-degree and thirty-degree endoscopes.

### Assessment of surgical trajectories

A non-contrast head CT scan was performed for preoperative planning of the ESTAH approach in our cadaveric specimens. Measurements of the tentorial angle and the distance between the posterior fossa dura mater at the potential craniotomy site and the uncus of the hippocampus were obtained using the Weasis DICOM medical viewer (version 4.1.1, 2023). These measurements were derived from the CT scans of each specimen to optimize surgical planning and anatomical assessment.

## Results

### Microsurgical white matter fiber dissection

**Fig. 1:**
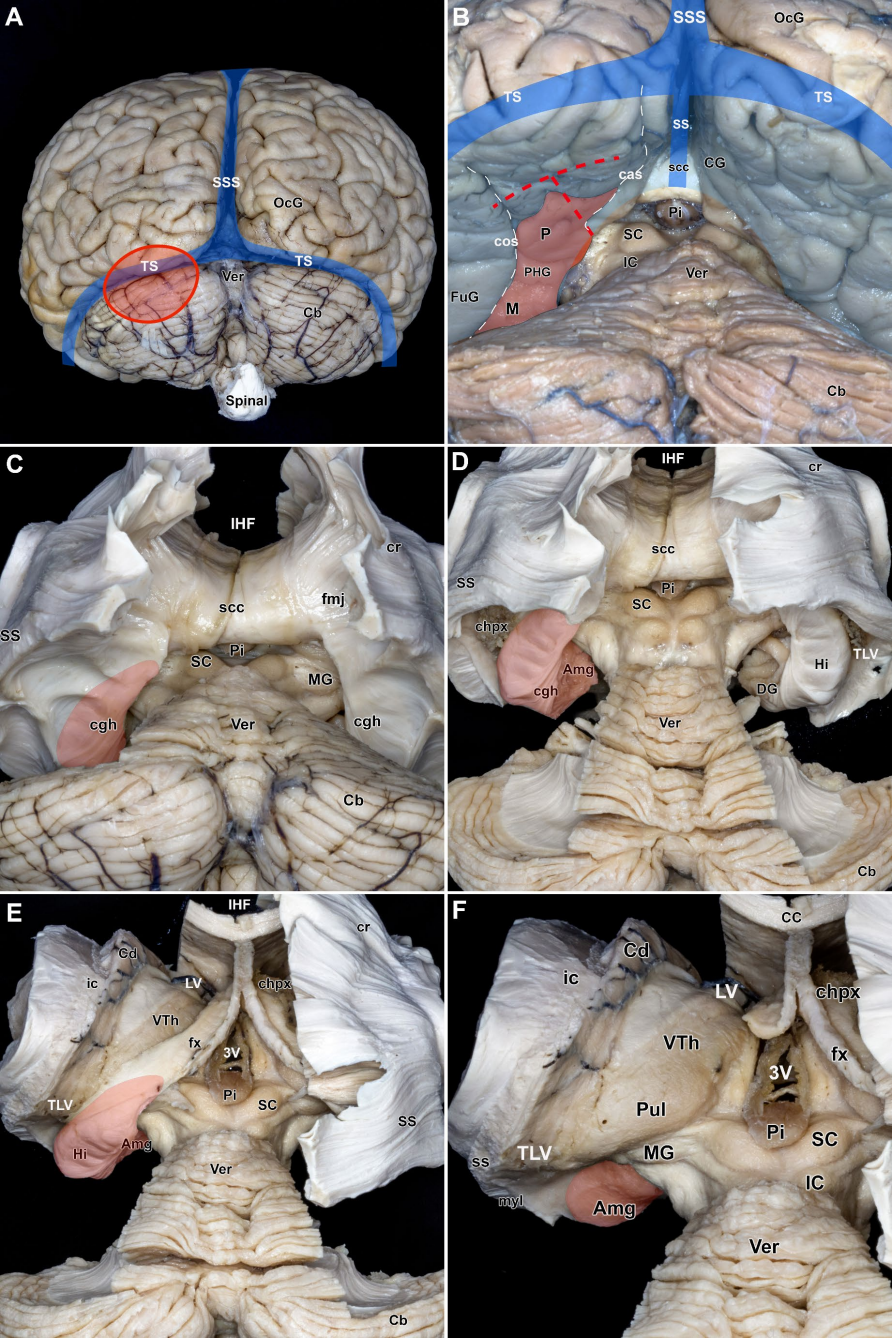
White Matter Microscopic Fiber Dissection. **A**: Posterior view of the superficial brain anatomy following the removal of vessels, arachnoidal, and pial membranes. The dural venous sinuses are highlighted in blue, and the area unsuitable for the ESTAH approach is marked in red. **B**: Opened supracerebellar space with the mediobasal temporal region (MTR) exposed, showing key anatomical landmarks for the ESTAH approach. The left-sided parahippocampal gyrus is highlighted in red. The white dashlines indicate the trajectory of the collateral and calcarine sulci. **C**: Following decortication and the removal of U-fibers, the thin cingulum fibers over the MTR are exposed. The orientation of the left-sided hippocampus is highlighted. **D**: After removal of the cingulum fibers, the hippocampus, with the amygdala anterior to it, and the temporal horn of the lateral ventricle (TLV) laterally, are exposed. The left-sided hippocampus and amygdala are highlighted. **E**: The left-sided corona radiata fibers are removed, and the corpus callosum is transected to expose the entire lateral ventricle, allowing the identification of the fimbria/fornix and the trajectory of the caudate nucleus and their relationship to the hippocampus. The left-sided hippocampus and amygdala are highlighted. **F**: Close-up view following the removal of the left-sided hippocampus. The dissection reveals the thalamic pulvinar superiorly, the medial geniculate body, and the amygdala anteriorly, noted for its nutmeg-like appearance. The amygdala is highlighted. 3 V - third ventricle; Amg - amygdala; cas - calcarine sulcus; Cb - cerebellum; CC - corpus callosum; Cd - caudate nucleus; CG - cingulate gyrus; chpx - choroid plexus of the lateral ventricle; cos - Collateral sulcus; cgh - hippocampal part of the cingulum; cr - corona radiata; fx - fornix; fmj - forceps major; FuG - fusiform gyrus; Hi - hippocampus; IC - inferior colliculus; IHF - interhemispheric fissure; LV - lateral ventricle; M - middle portion of the MTR; MG - medial geniculate body; myl - Meyer’s loop; OcG - occipital gyrus; P - posterior portion of the MTR; PHG - parahippocampal gyrus; Pi - pineal gland; Pul - pulvinar; SC - superior colliculus; scc - splenium of the corpus callosum; SSS - superior sagittal sinus; White SS - straights sinus; Black SS - sagittal stratum; Spinal - spinal cord; TS - transverse sinus; TLV - temporal horn of the lateral ventricle; Ver - vermis; VTh - velar portion of the thalamus; Highlighted with red resection area for amygdalahippocamectomy. The red-highlighted areas indicate the resection zone for amygdalohippocampectomy

Following fixation and a detailed examination of the surface anatomy of the brain, white matter dissection was performed (Fig. 1A). The supracerebellar corridor provides access to the posterior and middle portion of the MTR, as well as the anterior portion through its middle portion. The MTR is composed of the amygdala, uncus, parahippocampal gyrus, and hippocampus. It is bordered laterally by the collateral sulcus and surrounded by vital neural and vascular structures, including the optic tract, lateral geniculate body, oculomotor and trochlear nerves, midbrain, internal carotid artery, anterior choroidal artery, and posterior cerebral artery, as well as the basal vein of Rosenthal medially (Fig. 1B).

The procedure of amygdalohippocampectomy begins with the resection of the parahippocampal gyrus at the level of the inferior colliculus (anterior splenial line). Removal of the gray matter of the parahippocampal gyrus exposes poorly developed short association fibers (U-fibers). Following this, a thin layer of cingulate fibers is exposed (Fig. 1C). The cingulum, consisting of short and long associative fibers, surrounds the corpus callosum, passing through the cingulate gyrus and parahippocampal gyrus, extending from the septal area to the uncal region of the MTR. Removal of the cingulate fibers reveals the amygdala, hippocampus, and the temporal horn of the lateral ventricle (TLV), which contains the choroid plexus and is situated lateral to the hippocampus (Fig. 1D).

The fornix, a large arc-shaped bundle originating from the superior surface of the hippocampus, projects into the septal region and hypothalamus (Fig. 1E). Its terminal segment, the columna fornicis, passes through the hypothalamic wall and terminates in the mammillary body. The fornix is dissected from the tail of the hippocampus, and the hippocampal body and head are resected in piecemeal fashion (Fig. 1F). This allows exposure of the thalamic pulvinar and the tail of the caudate nucleus superiorly, the TLV laterally, and the amygdala anteriorly. At this step, the amygdala is readily apparent and discernible by its nutmeg-like coloration beneath the ependymal layer (Fig. 1F). The resection of the amygdala from this angle allows for the controlled resection and preservation of the Meyer’s loop.

### ESTAH technique

**Fig. 2:**
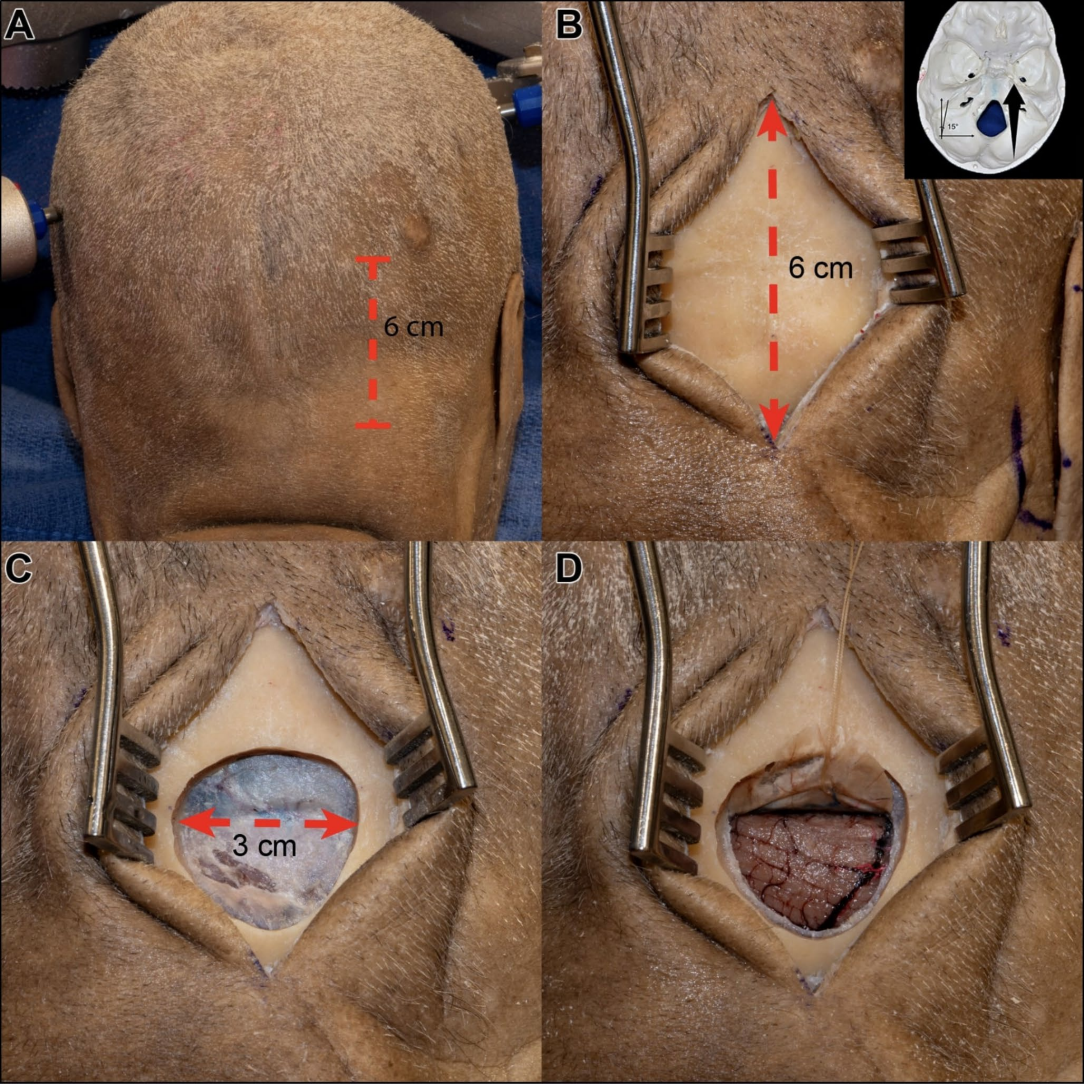
Dural Incision and Craniotomy. **A**: The head was positioned with a 15-degree ipsilateral angulation to provide a direct surgical corridor to the MTR. **B**: A straight 6 cm incision was made over the paramedian suboccipital region to facilitate the ESTAH approach. **C**: A circular 3 cm craniotomy was performed, exposing the lower half of the transverse sinus. **D**: A C-shaped incision of the dura mater was made and reflected superiorly, enhancing access to the supracerebellar space

The cadaveric heads were positioned in the modified Concorde (prone oblique) position and secured with a three-pin cranial fixation device (Mayfield^®^ clamp model). Two ipsilateral pins were placed in the contralateral mastoid region, while the contralateral pin was fixed at the superior ipsilateral temporal line above the temporal muscle (Fig. 2A). The heads were rotated 15 degrees towards the contralateral side to allow direct visualization of the MTR. A 6 cm longitudinal incision was made along the midline, between the inion and the asterion, encompassing approximately one-third of the occipital region and two-thirds of the suboccipital region (Fig. 2B). The target bone area was exposed using a self-retaining retractor. A single burr hole was drilled over the superior nuchal line, located in the upper aspect. A dural dissector was then used to detach the dura mater and transverse sinus from the bone. A circular bone flap was created with a craniotome, resulting in a 3 cm craniotomy that exposed the lower part of the transverse sinus (Fig. 2C). The dura mater was incised in an inverted C-shape, allowing for superior retraction of the flap, which was then positioned over the transverse sinus (Fig. 2D).

**Fig. 3:**
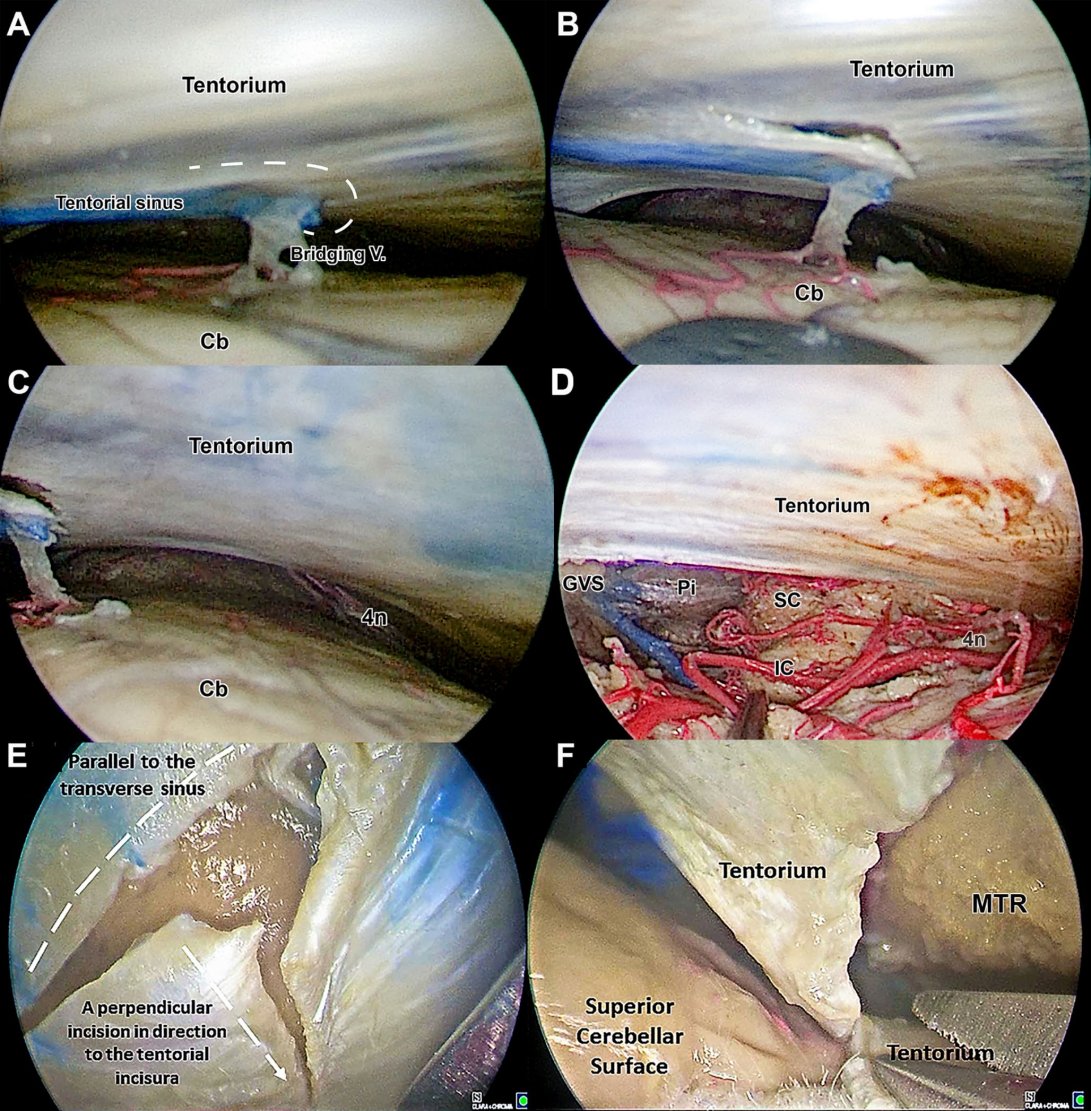
Supracerebellar Space Exploration. **A**: A primary endoscopic examination was performed using a zero-degree endoscope to explore the supracerebellar space, identifying bridging veins draining into the tentorial sinus. Careful management was required due to the midline orientation of the tentorial sinus. A right-sided C-shaped incision (White dashes) of the tentorium was made to preserve the bridging veins while ensuring adequate access to the surgical field. **B**: The tentorial incision reduced tension on the bridging veins, facilitating a safer and more expansive surgical corridor into the supracerebellar space. **C-D**: As the surgical corridor deepened, the Galenic venous system, pineal gland, superior and inferior colliculi, and the trochlear nerve were identified. **E**: A T-shaped incision of the tentorium was performed. The first cut was made parallel to the transverse sinus, and the second, perpendicular, extended to the tentorial incisura. **F**: The final segment of the tentorium was cut under endoscopic visualization, enhancing procedural safety. 4n - trochlear nerve; Cb - cerebellum; IC - inferior colliculus; MTR - mediobasal temporal region; Pi - pineal gland; SC - superior colliculus; The white dashed lines indicate the locations of dural incisions

At this stage, a zero-degree endoscope was introduced into the dissection field, providing a magnified view (Fig. 3A). The ipsilateral supracerebellar space was accessed, and several small tentorial draining veins were identified and preserved using the tentorial cut technique (Fig. 3B). A thorough inspection of the supracerebellar space up to the tentorial incisure was conducted. The thick arachnoid layer covering the posterior aspect of the quadrigeminal cistern, and the ambient cistern was identified and carefully dissected (Fig. 3C). This maneuver allowed for the visualization of critical neurovascular structures, such as the trochlear nerve, superior and inferior colliculi, pineal gland, and the Galenic venous system (Fig. 3D).

**Fig. 4:**
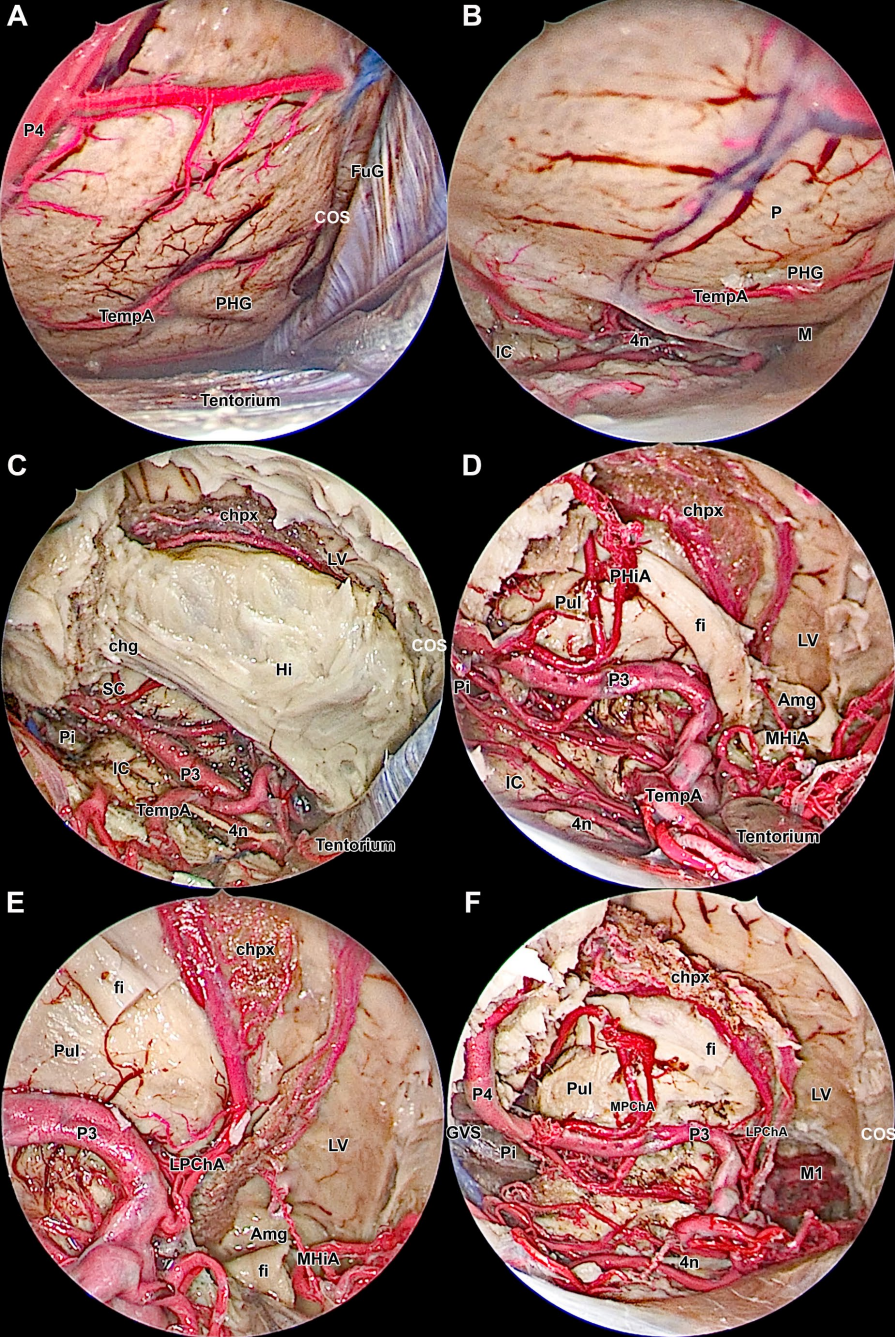
Anatomy and Technique of Endoscopic Amygdalohippocampectomy. **A**: Using a zero-degree endoscope, the anatomy of the mediobasal temporal region (MTR) was studied. The fusiform gyrus, collateral sulcus, and parahippocampal gyrus were identified. **B**: The anterior splenial line was identified. **C**: After decorticating the parahippocampal gyrus and removing the “U” fibers, the hippocampus and temporal horn of the lateral ventricle (TLV) were exposed. **D**: Removing the hippocampus allowed for the visualization of the fimbria, choroidal plexus, and branches of the posterior cerebral artery. **E**: Cutting the fimbria exposed the thalamic pulvinar, uncal recess, and the amygdalar complex. **F**: A general 30-degree endoscopic view of the surgical corridor after the amygdalohippocampectomy. 4n - trochlear nerve; Amg - amygdala; cgh - hippocampal part of the cingulum; chpx - choroid plexus of the lateral ventricle; COS - collateral sulcus; fi - fimbria; FuG - fusiform gyrus; Hi - hippocampus; IC - inferior colliculus; IC - internal carotid artery; LPChA - lateral posterior choroidal artery; LV - lateral ventricle; M - middle portion of the mediobasal temporal region (MTR); MHiA - middle hippocampal artery; MPChA - middle posterior choroidal artery; P - posterior portion of the MTR; P3 - P3 segment of the posterior cerebral artery; P4 - P4 segment of the posterior cerebral artery; PHG - parahippocampal gyrus; PHIA - posterior hippocampal artery; Pi - pineal gland; Pul - pulvinar; SC - superior colliculus; TempA - temporal artery; Tentorium - tentorial surface

The tentorium was incised in a T-shape, with the longitudinal incision made near the transverse sinus up to the tentorial incisure. The transverse incision was made parallel and near the transverse sinus with the aim of exposing the MTR (Fig. 3E, F). The dissection continued with the identification of the fusiform gyrus, lingual gyrus, parahippocampal gyrus, and collateral sulcus (Fig. 4A, B). All structures were confirmed using the neuronavigation system.

The anterior splenial line was utilized as a central reference point to direct the dissection, while the collateral sulcus served as the lateral boundary (Fig. 4A). The dissection proceeded with the removal of the cortex of the parahippocampal gyrus (Fig. 4B). Following decortication and the removal of weakly developed short associated “U” fibers, the hippocampus overlaid by callosal fibers, was exposed. Subsequently, the TLV was opened in a lateral position relative to the hippocampus (Fig. 4C).

The hippocampus was gently excised, thereby exposing the fimbria of the hippocampus along with the posterior and middle hippocampal arteries (Fig. 4D). The fimbria was sharply divided and separated from the thalamic pulvinar and choroidal plexus, allowing for clear visualization of the medial and lateral posterior choroidal arteries. Within the surgical corridor, the amygdalar nucleus and uncal recess were identified in anterosuperior position (Fig. 4E).

**Fig. 5:**
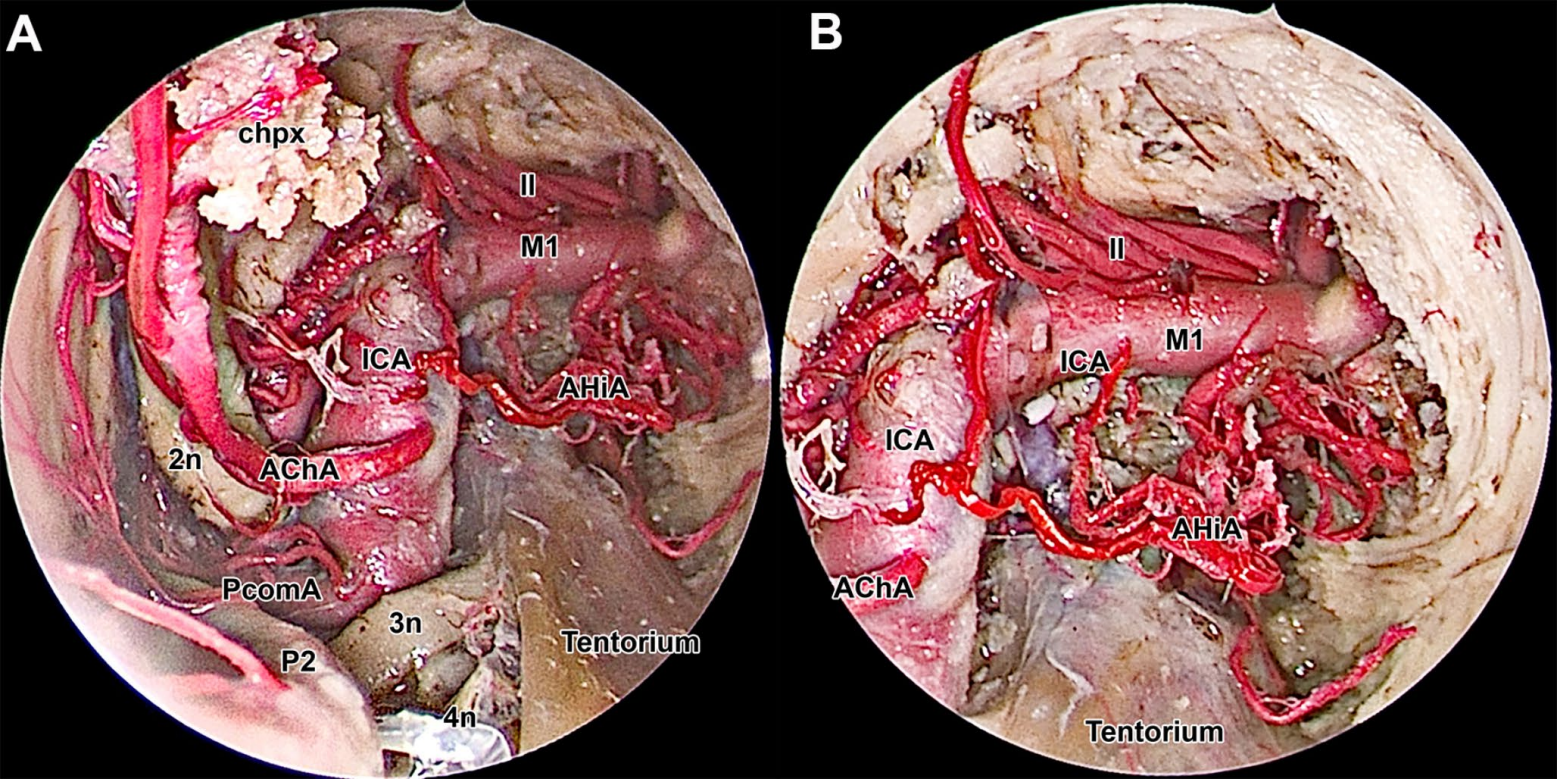
Close-Up Endoscopic View of the Anterior Part of the Surgical Corridor Following Endoscopic Amygdalohippocampectomy. **A**: Anteromedial view of the final stage, involving dissection and resection of the temporal pole. Key anatomical structures delineating the dissection boundaries are identified, including the internal carotid artery (ICA), anterior choroidal artery (AChA), branches of the middle cerebral artery (MCA), and cranial nerves II (optic nerve), III (oculomotor nerve), and IV (trochlear nerve). **B**: Anterior limit of dissection, highlighting critical neurovascular structures. 2n - optic nerve; 3n - oculomotor nerve; 4n - trochlear nerve; AChA - anterior choroidal artery; AHiA - anterior hippocampal artery; chpx - choroid plexus of the lateral ventricle; ICA - internal carotid artery; ll - lenticulostriate arteries; M1 - first segment of the middle cerebral artery; P2 - second segment of the posterior cerebral artery; PcomA - posterior communicating artery

The amygdalar nucleus was dissected using both zero-degree and 30-degree endoscopes to optimize visualization and precision. Resection of the amygdalar nucleus includes removal of the uncus and piriform cortex, with the superior and lateral boundaries are marked by the roof of the temporal horn of the lateral ventricle. The resection process allows for clear exposure of the internal carotid artery, anterior choroidal artery, posterior communicating artery, middle cerebral artery, optic nerve, oculomotor nerve, and trochlear nerve (Figs. 4F and 5A and B).

At the conclusion of the subpial amygdalohippocampectomy, the tentorial flaps were approximated with a simple stitch.

### Key anatomical landmarks

In all specimens, the trochlear nerve served as a key anatomical landmark to facilitate the identification of the tentorial incision, collateral sulcus, and parahippocampal gyrus in a safe and accurate manner (Figs. 1B, 3C and D and 4A and B). The ipsilateral inferior colliculus was used to identify the anterior splenial line, which served as the initial point for dissection of the parahippocampal gyrus. Once access to the TLV was achieved, the roof of the TLV was established as the lateral boundary of the dissection (Figs. 1D-F and 4C-F). Subsequently, the dissection advanced medially to the collateral sulcus, encompassing the body and head of the hippocampus, the parahippocampal gyrus, the fimbria, the subiculum, and the amygdaloid complex, until the middle cerebral artery was exposed anteriorly and the internal carotid artery anteromedially (Figs. 1D-F and 5A-B).

### Anatomical measurements and observations

**Fig. 6:**
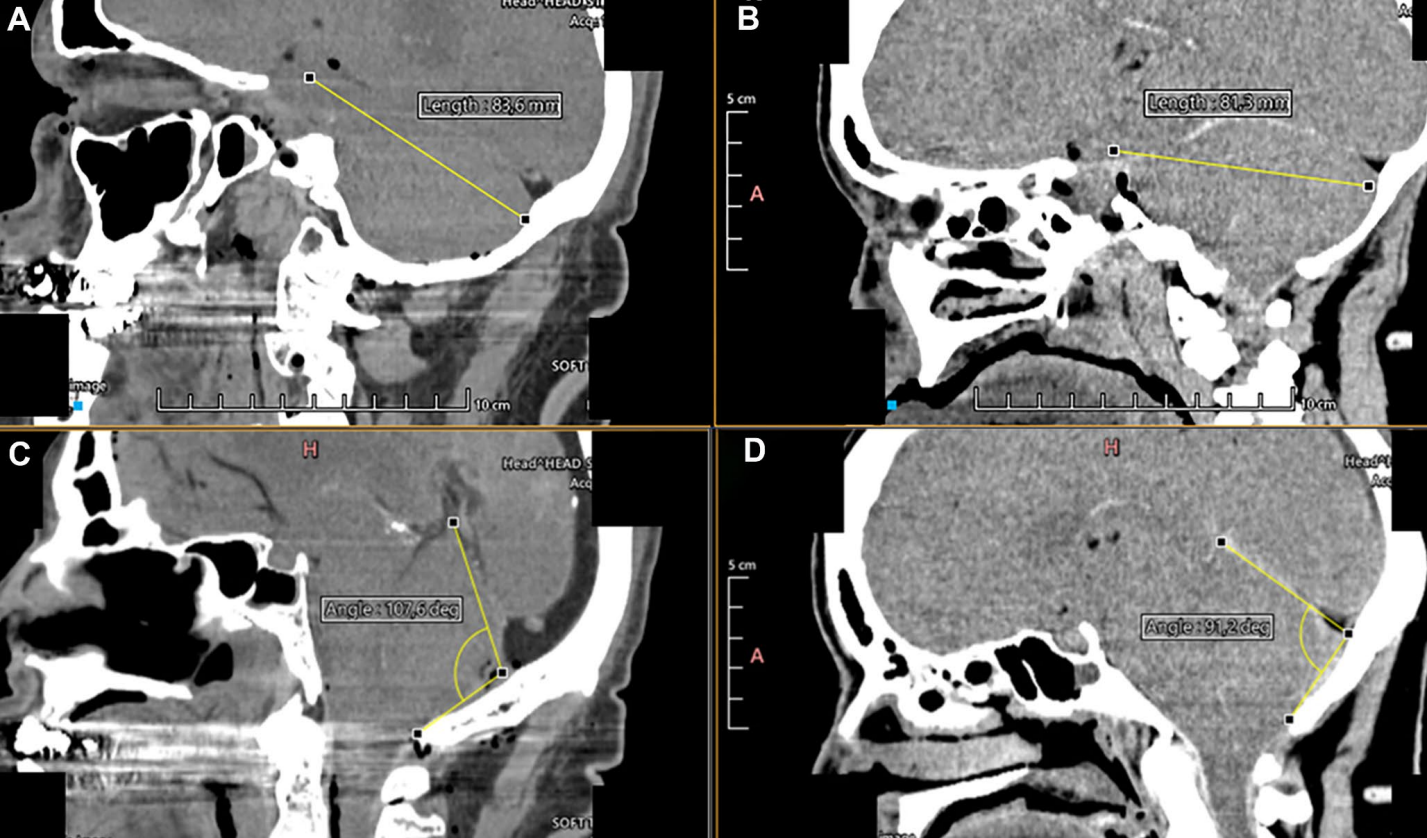
Anatomical Measurements and Observations. **A**,** B**: The analysis was conducted using four cadaveric specimens. A straight line was established between the dura mater of the posterior fossa and the uncus of the hippocampus. The reference point on the dura mater was found to be approximately 10 mm below the transverse sinus, and this trajectory mimics the endoscopic corridor. The average distance measured along this trajectory was 78.15 mm, with a range from 72 mm to 83.6 mm. **C**,** D**: The tentorial angle was assessed in the same four cadaveric specimens. This angle was defined as the intersection between a line connecting the Torcula Herophili and the Opisthion and the angle of the straight sinus. The average tentorial angle recorded was 104.1 degrees, with measurements ranging from 91.2 degrees to 110.9 degrees. This methodology differs from traditional tentorial angle measurements, which typically involve a line connecting the Nasion and the tuberculum sellae in relation to the angle of the straight sinus

Preoperative anatomic assessments were performed using non-contrast head CT scans of the cadaveric specimens. The hippocampal uncus was identified in axial, sagittal, and coronal views to determine its spatial relationship to the posterior fossa dura mater at the planned craniotomy site. The anteroposterior distance between these structures was measured to determine the depth of the surgical corridor, which is critical for selecting appropriate instrumentation and optimizing maneuverability. The mean distance measured was 78.15 mm (range: 72–83.6 mm) (Fig. 6A, B).

In addition, we measured the tentorial angle, defined as the intersection of a line connecting the torcula herophili and opisthion and the axis of the straight sinus. This angle reflects the inclination of the tentorium and directly influences the working trajectory of the endoscope and surgical instruments. The mean tentorial angle was 104.1° (range: 91.2°-110.9°) (Fig. 6C, D). Despite the variability in this parameter between specimens, no technical difficulties were encountered during the tentorial incision, as the endoscopic approach allowed precise adjustments in visualization and instrument angulation.

## Discussion

**Fig. 7:**
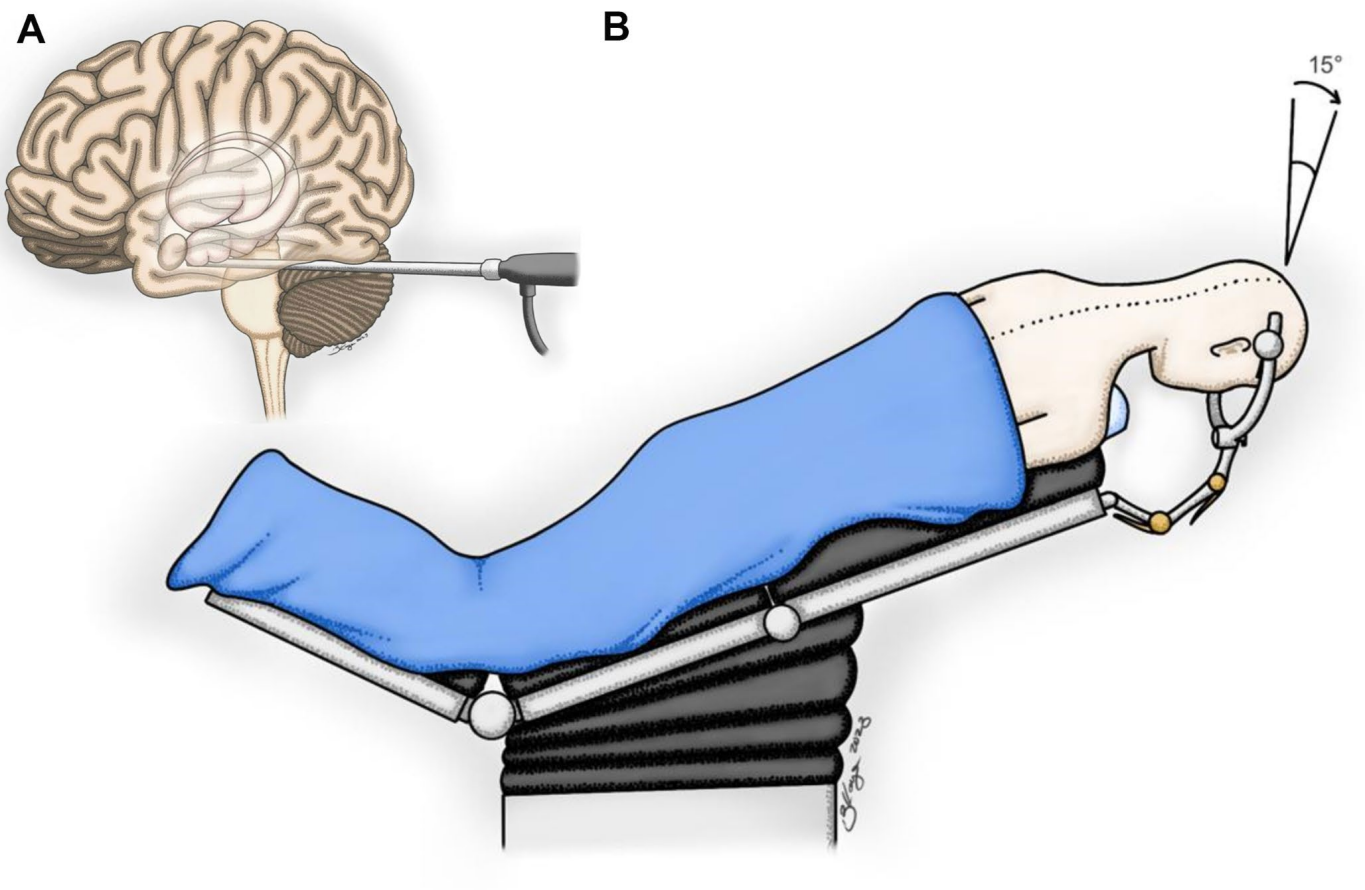
ESTAH Approach and Modified Concorde (Prone Oblique) Position. **A**: This illustration depicts the ESTAH approach. The primary advantage of this technique is the magnification of the surgical field provided by the endoscope in a posterior-anterior view, allowing access to the entire parahippocampal gyrus without violating adjacent structures. **B**: This illustration demonstrates the proposed modified Concorde position for ESTAH surgery. In this position, the patient’s head is rotated 15º to 30º contralateral to the surgical side relative to the floor. This maneuver ensures that the endoscope is positioned directly over the amygdaloid body

This anatomical study explores the feasibility of the ESTAH approach, carefully evaluating the critical landmarks surrounding the region and assessing the associated risks. Positioned as an evolution beyond the microscopic supracerebellar transtentorial approach, it aims to incorporate the principles of keyhole surgery through endoscopic techniques. Unique in its approach, this study establishes a standardized, step-by-step dissection pathway for MTR structures via a transtentorial, purely endoscopic route (Fig. 7A). This technique is suitable for various procedures, including selective amygdalohippocampectomy for epilepsy and resection of various lesions in this region.

### Positioning

In MTR surgery, two well-established positions are often discussed in the literature: the semi-sitting position and the modified park bench position. In the semi-sitting position, the head remains in a neutral position, perpendicular to the floor, with the legs semi-flexed and parallel to the head [9, 13]. This position offers excellent anatomical orientation and benefits from gravitational retraction; however, it requires careful monitoring for air embolism and limits the use of intraoperative MRI [9, 13, 17].

The modified park bench position places the patient in a lateral or three-quarter supine position with the head turned to the opposite side to expose the paramedian supracerebellar region, followed by slight flexion [14–16]. 

In our study, we introduce a third method of positioning, the modified Concorde (prone oblique) position (Fig. 7B), where the cadaver’s head is placed in a prone oblique position, rotated 15 to 30 degrees contralaterally. This maneuver positions the endoscope directly over the amygdaloid body, optimizing the view for dissection. This approach is intended to reduce the risks associated with the modified park bench and semi-sitting positions while maintaining adequate anatomical exposure. In addition, the modified Concorde position enhances alignment of deep-seated structures by improving the tentorial angle, which may allow for better surgical access and visualization [18]. 

### Incision and craniotomy

Using an endoscopic technique, brain relaxation can be achieved by draining cerebrospinal fluid (CSF) from the supracerebellar space, quadrigeminal cistern, or cerebellopontine angle, bypassing the traditional foramen magnum route. Based on this approach, we propose a 6.0 cm incision that provides adequate access while minimizing tissue disruption, in contrast to the 12 cm incision recommended in previous studies [9, 13]. Our dissections resulted in a mean craniotomy of 3.0 cm × 3.0 cm with a single burr hole over the transverse sinus (Fig. 2B, C), requiring exposure of only its inferior portion, thus ensuring a more precise and less invasive approach.

### Dural opening and the supracerebellar space

After the dural incision and access to the supracerebellar space, the tentorial bridging veins become clearly visible. While the microsurgical PST approach with its wider craniotomy improves visualization of the supracerebellar space and creates a surgical corridor between the tentorial bridging veins, our technique focuses on preserving these veins. The tentorial incision method, which was developed to preserve the bridging veins in the microscopic approaches, can also be effectively used in ESTAH surgery (Fig. 3A, B). This method ensures the preservation of the tentorial bridging veins without causing damage or complications, thereby minimizing the risk of cerebellar infarction and hemorrhage [14, 19, 20]. In addition, the enhanced endoscopic view allows for a smaller craniotomy, helping to preserve a greater number of these critical veins [21]. 

The ESTAH approach also safely identifies and dissects neurovascular structures within the quadrigeminal and surrounding cisterns, such as the pineal gland, vein of Galen, basal vein of Rosenthal, trochlear nerve, and distal branches of the anterior and posterior vascular circuits (Fig. 3C, D). Given the significant thickness of the subarachnoid membrane in this region, the endoscope plays a critical role in ensuring safe and precise dissection [21–23]. 

### Tentorial opening

While some neurosurgeons prefer a V-shaped incision over the tentorium, securing a suture at the tentorial flap [13], others advocate for tentorial resection to expose the entire mediobasal aspect of the inferior temporal lobe [24]. Drawing from our anatomical study, we propose a T-shaped incision over the tentorium, in line with the technique outlined by Teton et al. (Fig. 3E, F) [25]. Additionally, we underscore the precision and safety afforded by the endoscopic view during incision. Furthermore, the ESTAH approach facilitates the identification and safe resection of the tentorium’s free edge, mitigating risks to the trochlear nerve. Still, it is fundamental to take into account tentorial vasculature and its variations; namely a meningeal artery supplying the medial tentorium, arising from the posterior cerebral artery (PCA), the artery of Davidoff and Schechter (ADS), as well as the more conspicuous tentorial artery (or artery of Bernasconi & Cassinari) [21, 22]. 

### Amygdalohippocampectomy

The meticulous maneuvering of an amygdalohippocampectomy is revealed through the opening of the tentorium, which presents a view of neurovascular structures. Each of these structures plays a crucial role in the surgical nuances of this approach. At the nexus of precision and purpose, lies the parahippocampal gyrus, which is significant due to its association with the inferior colliculus. As the surgical procedure progresses, a sequential resection is performed, tracing the delicate contours of the parahippocampal gyrus, the surface nuances of the hippocampus body/head, and finally, the intricate depths of the amygdala (Figs. 1B-F and 4A-F) [26–31]. 

The boundaries of the surgical space are established, comprising the collateral sulcus, the lateral limit, and the choroidal fissure and midbrain, which represent the medial confines [9]. In this meticulous undertaking, the neuronavigation system serves as a reliable aid, guaranteeing that the trajectory and distance remain aligned with the surgical objective.

Two potential pathways present themselves: a progressive resection of the parahippocampal gyrus towards the hippocampal body/head, or a meandering course following the collateral sulcus to the TLV. In this delicate maneuvering, the choroidal fissure plays a crucial role. Its posterior-to-anterior dissection marks a necessary passage (Fig. 5A, B) [9, 12, 32]. 

A comprehensive understanding of white matter fiber tracts, including the “U” fibers, cingulum, fimbria, and their spatial relationships with adjacent structures like the sagittal stratum, is essential for precise dissection during amygdalohippocampectomy (Fig. 1A-F) [26–31, 33]. The sagittal stratum, situated within the temporal and occipital lobes, comprises the optic radiations (including Meyer’s loop) and tracts that facilitate connectivity between the occipital lobe and the hippocampus and parahippocampal regions (Fig. 1C, D) [34, 35, 36]. Injury to this structure can result in substantial deficits in visual field function. The supracerebellar transtentorial approach allows surgeons to better preserve the integrity of the optic radiation, minimizing collateral damage and optimizing neurological outcomes in comparison to lateral approaches [9, 13, 33, 36–39]. 

Navigating the depths of the brain through a posterior approach poses its own set of challenges, notably in identifying elusive structures such as the amygdala and temporal pole beyond the uncal apex encircling the cerebral peduncle, as highlighted by Yasargil [4]. Likewise, it is deeply stressed and reaffirmed that the respective vasculature supplying this set of landmarks and its anatomy cannot be overlooked: the Anterior Choroidal Artery along with the hippocampal branch of the PCA provide nourishment to the amygdala and a crucial portion of the MTR [40]. The uncus, dentate gyrus, subiculum and Ammon’s horn conform altogether both the perirhinal and entorhinal cortex, jointly establishing what is known as the parahippocampal gyrus [22, 23, 26, 27, 31]. Notably, an anastomosis between the anterior temporal branch of the PCA and the anterior temporal branch of the MCA is often found in the anterior third of the temporal lobe [21, 28, 29]. Yet, the advent of endoscopic techniques offers a transformative solution, affording surgeons a magnified view and enhanced visualization in these critical regions. The incorporation of angled endoscopes, such as the 30-degree endoscope in our dissections, has notably enhanced our surgical precision and outcomes.

During dissection, exposing the PCA perforators is critical in clinical settings to maintain subpial dissection, averting catastrophic outcomes [21, 26, 27, 31]. It’s imperative to identify and preserve all landmarks meticulously: (a) the choroidal fissure with the anterior choroidal artery, optic tract, and midbrain with the PCA medially; (b) the collateral sulcus and rhinal sulcus directing to the TLV; (c) the temporal roof superiorly; and (d) the oculomotor nerve, the ICA, and the MCA branches anteriorly (Fig. 5A-B) [23, 26, 27, 31]. 

The relationship between the transverse sinus and the petrous ridge poses challenges to the inferior and anterior aspects of the parahippocampal gyrus. Türe et al. propose a solution: placing cotton sponges in the middle fossa to expose these structures, aiding in parahippocampal gyrus resection [9]. Our dissections encountered no difficulty in this aspect, possibly because the 30-degree endoscope facilitated an angled view of the neurostructures.

When considering the role of the temporal pole in epileptogenesis, there’s substantial evidence linking it to the amygdala-hippocampus-parahippocampal complex, particularly evident during seizures’ early consciousness impairment [39]. Building on this premise, our dissections targeted the temporal pole, revealing a challenging, deep, and narrow corridor, leading to heightened tissue manipulation. Consequently, for cases planning temporal pole resection, we advise against this approach, favoring an *en bloc* anterior temporal lobectomy through a traditional anterior approach [41–45]. 

The ESTAH approach ensures safe hemostasis by providing a comprehensive analysis of the surgical field through the endoscopic view, which includes both zero-degree and thirty-degree endoscopes [11]. This eliminates the blind spot often seen with microscopic approaches. Although it’s not mandatory to reapproximate the dural flaps, we advocate doing so to prevent herniation of occipital structures.

### Strengths and limitations

While the ESTAH approach shows considerable promise, it has a number of limitations. It is imperative that practitioners possess both expertise in endoscopic techniques and a comprehensive understanding of the anatomy of the inferior MTR. The enhanced visualization provided by the endoscopic view can, on occasion, lack a panoramic perspective, which may result in inaccurate assessments of critical neurovascular structures. It is recommended that neuronavigation be employed in order to enhance accuracy.

The utilisation of a pneumatic holding arm permits dual-handed operation; however, it does so in a confined space, thereby presenting certain challenges. The use of long instruments is imperative, particularly in the context of amygdala and hippocampal head resections. Although 4 mm endoscopes demonstrated satisfactory performance, it remains uncertain whether they are more effective than neuroendoscopes utilized in third ventriculostomies. The study’s reliance on a limited number of cadaveric specimens restricts the scope for exploring anatomical variations.

Furthermore, the use of formalin-fixed, latex-injected cadaveric heads introduces artifacts and does not fully replicate the tissue properties observed in vivo. The specimens lack the physiological dynamics and surgical complexities observed in MTR tumors or sclerosis, which limits their applicability.

It is imperative that this approach undergoes clinical validation to confirm its utility. Furthermore, it is of paramount importance that the skull base laboratory continues to develop the requisite skills. Notwithstanding these limitations, the ESTAH approach demonstrates promise as a potential alternative technique for addressing this pathology.

## Conclusion

The landscape of minimally invasive neurosurgery for the MTR continues to evolve, and this study adds valuable insights to the ongoing discourse by delineating the merits and drawbacks of the ESTAH approach. Offering a direct corridor to the focal area minus unnecessary corticotomy or brain retractions, this approach facilitates magnified visualization and precision in dissections, even with a smaller craniotomy. Consequently, the theoretical risk of complications associated with this technique is lower. Moreover, it aids in circumventing pitfalls linked to traditional approaches, such as visual field impairments and neuropsychological sequelae. We believe our work strives to shed valuable light on the matter.

## Data Availability

No datasets were generated or analysed during the current study.

## Abbreviations

PST: Paramedian supracerebellar–transtentorial approach
ESTAH: Endoscopic supracerebellar transtentorial approach for an amygdalohippocampectomy
MRT: Mediobasal temporal region
TLV: Temporal horn of the lateral ventricle

## Declarations

### Previous presentations

Oral presentation at the 2024 North American Skull Base Society (NASBS) 33rd Annual Meeting, Atlanta, GA, USA, Feb 16–18, 2024.
